# Interaction between NS1 and Cellular MAVS Contributes to NS1 Mitochondria Targeting

**DOI:** 10.3390/v13101909

**Published:** 2021-09-23

**Authors:** Yeu-Yang Tseng, Chih-Ying Kuan, Masaki Mibayashi, Chi-Jene Chen, Peter Palese, Randy A. Albrecht, Wei-Li Hsu

**Affiliations:** 1 Graduate Institute of Microbiology and Public Health, National Chung Hsing University, Taichung 402, Taiwan; yeuyang.tseng@unimelb.edu.au (Y.-Y.T.); co99020030@email.nchu.edu.tw (C.-Y.K.); 2 WHO Collaborating Centre for Reference and Research on Influenza, Royal Melbourne Hospital, Peter Doherty Institute for Infection and Immunity, Melbourne, VIC 3000, Australia; 3 Department of Infectious Diseases, Peter Doherty Institute for Infection and Immunity, University of Melbourne, Melbourne, VIC 3000, Australia; 4 Department of Microbiology, Icahn School of Medicine at Mount Sinai, New York, NY 10029, USA; masaki.mibayashi@mssm.edu (M.M.); chijune0807@gmail.com (C.-J.C.); peter.palese@mssm.edu (P.P.); randy.albrecht@mssm.edu (R.A.A.); 5 Department of Medicine, Icahn School of Medicine at Mount Sinai, New York, NY 10029, USA; 6 Global Health and Emerging Pathogens Institute, Icahn School of Medicine at Mount Sinai, New York, NY 10029, USA

**Keywords:** influenza A virus, NS1, MAVS, mitochondria

## Abstract

Influenza A virus nonstructural protein 1 (NS1) plays an important role in evading host innate immunity. NS1 inhibits interferon (IFN) responses via multiple mechanisms, including sequestering dsRNA and suppressing retinoic acid-inducible gene I (RIG-I) signaling by interacting with RIG-I and tripartite motif-containing protein 25 (TRIM25). In the current study, we demonstrated the mitochondrial localization of NS1 at the early stage of influenza virus infection. Since NS1 does not contain mitochondria-targeting signals, we suspected that there is an association between the NS1 and mitochondrial proteins. This hypothesis was tested by demonstrating the interaction of NS1 with mitochondrial antiviral-signaling protein (MAVS) in a RIG-I-independent manner. Importantly, the association with MAVS facilitated the mitochondrial localization of NS1 and thereby significantly impeded MAVS-mediated Type I IFN production.

## 1. Introduction

Nonstructural protein 1 (NS1), encoded by segment eight vRNA [1] of the influenza A virus (IAV), harbors versatile biological functions. The NS1 ranges from 215 to 237 amino acids due to sequence variations among influenza virus strains [2,3]. Two distinct functional domains of NS1 have been defined, namely, the N-terminal RNA-binding domain (residues 1–73) and C-terminal effector domain (residues 88-202). The N-terminal domain of NS1 is critical for the virus interaction with different types of RNA, such as double-stranded RNA (dsRNA), and the C-terminal domain has been shown to interact with several cellular proteins [4,5].

The NS1 mainly localizes to the nucleus due to an arginine-rich nuclear localization sequence (NLS), [6,7,8]. Sequence alignment analysis indicated that the human H1N1 viruses circulating during the 1940s acquired a 7-aa extension at the C-terminus, and this extension generates a nucleolar localization signal (NoLS) [9], which mediates the nuclear and nucleolar localization of the NS1 [10]. The NS1 was previously demonstrated to interact with nucleolin within the nucleolus [8]. NS1 also possesses a nuclear export signal [7], which mediates its Crm-1-independent nuclear export [11]. In addition to its intrinsic cellular localization signals, interactions of host cellular proteins with NS1 influence its subcellular distribution [3]. The cytoplasmic localization of a subpopulation of NS1 has been observed, and this localization is likely due to the association of cellular proteins or RNA binding to and masking the NLSs of NS1 [12]. Cellular importin-α can shuttle NS1 into the nucleus, where it localizes within nuclear dot 10 (ND10) structures [10,13].

Several studies have demonstrated that the dynamic interplay of NS1 with multiple host proteins results in different cellular responses upon IAV infection [3,14]. Among these biological activities, antagonism of the host Type I interferon response is the most well-studied function of NS1. For example, a recombinant virus containing a truncated form of NS1 induced a higher level of IFN in infected cells and replicated more efficiently in IFN-deficient cells than it did in control cells [15]. This process can be illustrated in a mechanistic way: NS1 suppresses IFN-β expression via a pre-transcriptional mechanism by which NS1 binds dsRNA and thus inhibits the activation of interferon regulatory factor (IRF) 3 and nuclear factor NF-κB signaling pathways [16,17]. Additionally, IAV NS1 downregulates gene expression post-transcriptionally by interacting with CPSF30 [18] and by inhibiting pre-mRNA splicing [19,20]. Furthermore, NS1 suppresses the functions of IFN-induced proteins through different mechanisms, such as inhibiting dsRNA-dependent protein kinase R (PKR) by directly interacting with and restraining 2’-5’-oligoadenylate synthetase (OAS) activation through the sequestration of dsRNA from 2′-5′ OAS [21,22]. For retinoic acid-inducible gene I (RIG-I) signaling, NS1 inhibits the ubiquitination of RIG-I, one of the cytosolic RIG-I-like receptors (RLRs) for nucleic acid sensing, which is critical for IFN induction [23]. The IAV NS1 protein has been shown to interact with RIG-I, which in turn inhibits the mitochondrial antiviral-signaling protein (MAVS, also known as IPS-1, Cardif and VISA)-mediated signaling pathway [24,25]. Later, several studies demonstrated that the function of tripartite motif-containing protein 25 (TRIM25), an E3 ubiquitin ligase required for RIG-I activation, is also inhibited by the NS1 protein in a species-specific manner [23,25].

In the current study, we observed the localization of NS1 within cytoplasmic compartments and the accumulation of NS1 at mitochondrial membranes during the early stage of IAV infection. We investigated the possible mechanism by which NS1 targets mitochondria and discuss the relevance of this interaction to NS1 functions.

## 2. Materials and Methods

### 2.1. Plasmids

The plasmid pCAGGS-NS1 encoding the full-length NS1 sequence of A/Puerto Rico/8/1934 (PR8), NS1 R38A/K41A, NS1 E96A/E97A, NS1 ∆1-73 (N), or NS1 ∆74-230 (C) was previously described [16,24,25]. Plasmid FLAG-NS1 was obtained by cloing IAV PR8 NS1 amplified by PCR using primers with *Nhe* I and *Xba* I sequences at each end (NheI-NS1-FLAG-PR8-F: 5’-GGCCGCTAGCATGGATCCAAACACT GTGTCAA-3’ and XbaI-NS1-FLAG-PR8-R: 5’-GGCCTCTAGATCAAACTTCTG ACCTAATTGT-3’) into p3xFLAG-CMV-14 vector (Sigma-Aldrich, Saint Louis, MO, USA) linearized with corresponding restriction enzymes.

Plasmids expressing HA-tagged wild-type MAVS and deletions of CARD, PRD, or transmembrane domain (TMD) (namely ΔCARD, ΔPRD and ΔTMD) were provided by P.P. To generate plasmids expressing human MAVS and its variants with deletion of TM domain tagged with an HA sequence at the C-terminus, the inserts were respectively amplified by PCR from human A549 cDNA using a primer set (NheI-MAVS-F: 5′-TCGA GCTAGC ATGCCGTTTGCTGAAGACA-3′ with XbaI-MAVS-R: 5′-ACTCTAGACTACCCGGGGGCATAATCTGGCACATCATAAGGGTAGTAGTGCAGACGCCGCCGGTACAG-3’ or XbaI-MAVS-TM-R: 5′-AC TCTAGACTACCCGGGGGCATAATCTGGCACATCATAAGGGTAGTAGTGCAGACGCCGCCGGTACAGAGGTGAGGGCCTGTGGCATGGC-3′). The cDNA of full-length MAVS or ΔTM was digested by *Nhe* I and *Xba* I enzymes at 37 °C for 1 h and then ligated with pcDNA3.1 plasmid digested with corresponding enzymes.

### 2.2. Cells and Virus

Madin-Darby canine kidney (MDCK), human embryonic kidney 293 cell line expressing large T antigen (293T), human alveolar basal epithelial cells (A549) cells, RIG-I knockout A549 cells (RIG-I^−/−^), and chicken DF1 cells were cultured in Dulbecco’s modified Eagle’s medium (DMEM; GIBCO, BRL, Life Technologies Corporation, Carlsbad, CA, USA), supplemented with 10% fetal bovine serum (FBS; GIBCO), 1.0 mM sodium pyruvate, and 1% penicillin–streptomycin. All cells were incubated at 37 °C with 5% CO_2_. RIG-I^−/−^ cells were gifts from Professor Martin Schlee (University of Bonn, Germany) and Professor Patrick Reading (University of Melbourne, Australia) [26]. Influenza virus (PR8 strain) or PR8 expressing NS1 with FLAG-tag sequences inserted at the nucleotide of 515 were kindly provided by Dr. Nicholas S. Heaton, Duke University, USA [27].

### 2.3. Western Blotting (WB)

Cell lysates were harvested in sample buffer and proteins were separated by sodium dodecyl sulfate-polyacrylamide gel electrophoresis (SDS-PAGE), followed by transfer to nitrocellulose membrane. After blocking in 5% skim milk in PBS at room temperature for 1 h, primary antibodies, including anti-FLAG (1:2500; Signalway Antibody, College Park, MD, USA), anti-HA (1:2500; Sigma, Saint Louis, MO, USA), anti-β-actin (1:1000; Signalway Antibody, College Park, MD, USA), Histon H3 (1:1000, Cell signalling Technology, Danvers, MA, USA), or TOM-20 (1:1000, Abcam, Cambridge, UK) were used to probe specific protein at 4 °C overnight. Next, the membranes were incubated with corresponding secondary antibody conjugated with horseradish peroxidase (HRP) and protein signals were detected by ImageQuant LAS 4000 (GE Heathcare, Uppsala, Sweden). The membrane was washed by PBS with 0.05% Tween 20 for three times between each step.

### 2.4. Transfection and Immunoprecipitation (IP)

Cells were seeded 1 day before transfection. Briefly, 2 μg of indicated plasmid was transfected by Lipofectamine 2000 (Invitrogen, Carlsbad, CA, USA), according to the manufacturer’s guide. At 24 h post-transfection, cells were harvested in lysis buffer (50 mM Tris [pH 7.4], 150 mM NaCl, 1 mM EDTA and 1% Triton X-100) and were kept on ice for 30 min, followed by centrifugation at 12,000 rpm for 10 min. One-tenth of the supernatant was saved and used as input control. The rest of the supernatant was incubated with anti-FLAG M2 affinity beads (Sigma-Aldrich) at 4 °C overnight. Beads were collected by centrifugation at 12,000 rpm and then washed with wash buffer (50 mM Tris [pH 7.4] and 150 mM NaCl) for five times. Lastly, proteins were stored in sample buffer for Western blotting analysis.

### 2.5. Immunofluorescent Assay (IFA)

Indicated cells were transfected with various NS1 or MAVS constructs for 24 h, followed by fixation with 3.75% formaldehyde at room temperature for 30 min and then permeabilization with 0.5% NP40 at room temperature for 10 min. Cells were incubated with anti-MAVS (1:200 dilution; Santa Cruz Biotechnology, Dallas, TX, USA), anti-NS1 (1:200 dilution, Genetex, Alton Pkwy Irvine, CA, USA), or anti-S tag antibody (1:200, Novagen, San Diego, CA, USA) for 1 h at room temperature and then incubated with secondary antibodies conjugated with Alexa Fluor 488 (1:5000 dilution, Invitrogen) or Alexa Fluor 594 (1:5000, Invitrogen) for one additional hour. Wash steps were performed between each step using PBS containing 1% FBS.

### 2.6. Reporter Assay for IFN-β Promoter Activity

The 293T cells were co-transfected with a firefly luciferase reporter driven by an IFN-β promoter, a pRL-TK Renilla luciferase reporter, the V5-tagged MAVS-expressing plasmid, and 3xFLAG-tagged PR8 NS1-expressing plasmid or empty vector. After 48 h post transfection, total cell lysates were collected for western blot analysis and also the Dual-luciferase reporter assay (Promega, Madison, WI, USA), according to the manufacturer’s protocol. The expression of firefly luciferase was represented as the IFN-β promoter activity and Renilla luciferase was used as the internal control.

### 2.7. ELISA for Measuring IFN-β Concentrations

The 293T cells were co-transfected with plasmids expressing MAVS and NS1 or empty vector. One day after transfection, the culture medium was harvested for measuring the concentration of IFN-β by the standard procedure of enzyme-linked immunosorbent assay (ELISA) using an anti-IFN-β antibody (PBL Biochemical Laboratories, Piscataway, NJ, USA).

## 3. Results

### 3.1. Mitochondrial Localization of NS1 and Its Interaction with MAVS

**Figure 1:**
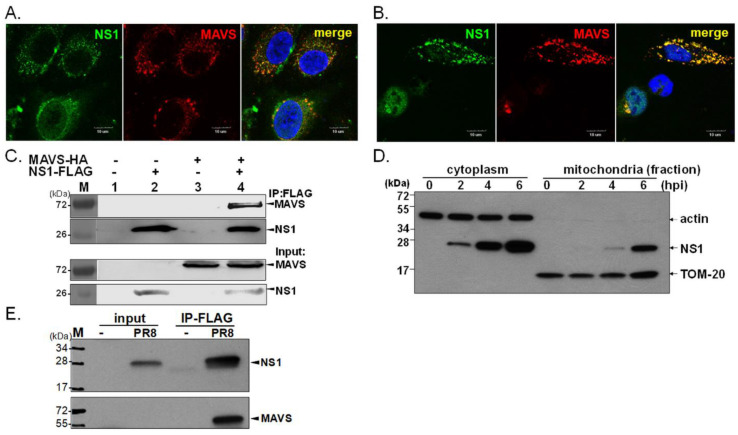
Colocalization of IAV NS1 and MAVS. Expression of NS1 in infected MDCK cells immunostained for endogenous MAVS and 4’,6-diamidino-2-phenylindole (DAPI) for nuclei (**A**). Human A549 cells were co-transfected with constructs expressing FLAG-NS1 and HA-MAVS to examine the cellular distribution and association of NS1 and MAVS by IFA (**B**) or IP assays (**C**). Human A549 cells were infected with IAV (PR8 strain) at a multiplicity of infection equal to 5. At 2, 4, and 6 hpi, cytoplasmic and mitochondrial fractions of infected cells were isolated by a Qproteome mitochondria isolation kit (Qiagen), and then Western blot analysis was performed to detect NS1, TOM-20 and actin; the latter two proteins indicate mitochondrial and cytoplasmic fractions, respectively (**D**). The interaction of endogenous MAVS with viral NS1 was also determined in A549 cells infected with a recombinant IAV expressing FLAG-tagged NS1 (**E**); cell lysates (input control) were harvested at 8 hpi, subjected to IP using FLAG antibody conjugated with beads and then WB for analysis of the interaction between FLAG-tagged NS1 and endogenous MAVS.

In an attempt to determine the dynamic cellular distribution of NS1, IFA was performed to determine the distribution of NS1 in two different cell types. In a subset of IAV-infected MDCK cells, a proportion of NS1 colocalized with endogenous MAVS (Figure 1A). Moreover, we assessed this observation in a transient expression system. Plasmid-expressed IAV NS1 accumulated within the nucleus or localized in a punctate pattern in the cytoplasm (Figure 1B). Of note, in some cells without overexpression of MAVS, the majority of NS1 remained nuclear (Figure 1B). Consistently, the punctate distribution of NS1 within the cytoplasm colocalized with mitochondrial MAVS protein in approximately 71% of the transfected A549 cells expressing both NS1 and MAVS (Figure 1B, and supplementary Figure S1). We further estimated the proportion of NS1 that colocalized with MAVS in individual cells by quantitative analysis by ImageJ and Olympus cellSens Dimension software (Version 1.1). Due to the dynamic cellular distribution of NS1, approximately 27.1–67% of NS1 protein localized with MAVS in cells co-expressing these two proteins based on ImageJ analysis and this finding was consistent with results measured by cellSens Dimension software (Table S1 and Figure S2). These statistical results indicated moderate level of colocalization between NS1 and MAVS and the level of such a colocalization was highly variable in different cells. In support of this observation, we detected by immunoprecipitation (IP) that overexpressed NS1 interacted with MAVS (Figure 1C), a finding consistent with our IFA results (Figure 1B,C). To examine if NS1 is localized to mitochondria during IAV infection, the localization of NS1 was investigated in IAV-infected human A549 cells. The infected cells were harvested at the indicated times, and then nuclear, cytoplasmic, and mitochondrial fractions were prepared for immunoblot assay. We detected a time-dependent increase in the amount of cytoplasmic and mitochondrial NS1 (Figure 1D). We further studied the interaction of endogenous MAVS with IAV NS1 during infection by a replication-competent reporter IAV expressing FLAG-tagged NS1 [27] (Figure 1E). Although NS1 itself does not contain mitochondrial targeting sequences, our data showed that IAV NS1 can localize to mitochondria, which may be facilitated, in part, by the association of NS1 with MAVS.

### 3.2. Identification of Domains of NS1 Required for the NS1-MAVS Interaction

**Figure 2:**
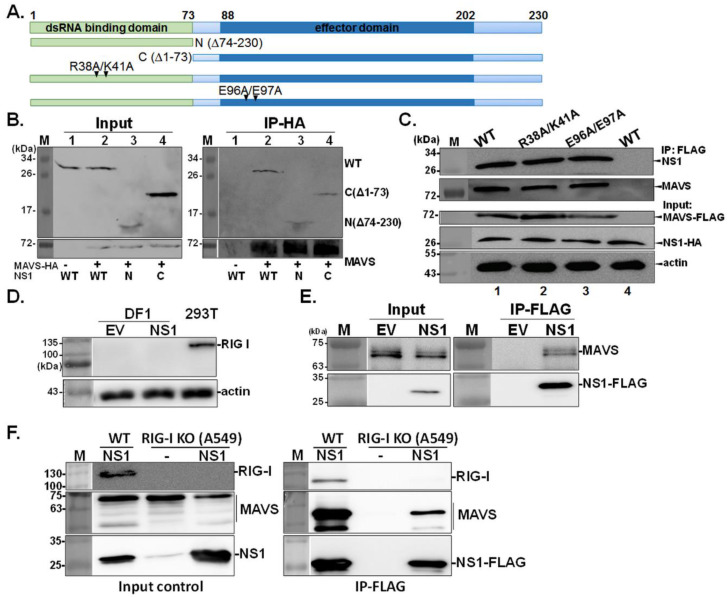
Domains of NS1 involved in the NS1-MAVS interaction. Four NS1-deletion constructs, namely, deletion of effector domain (N; Δ74–230), dsRNA-binding domain (C; Δ1–73), and two double-point mutations (R38A/K41A and E96A/E97A) at key residues participating in RIG-I and TRIM25 interaction, were used to study the domain involved in the NS1-MAVS interaction (**A**). The 293T cells were transfected with a HA-MAVS plasmid (Lane 1) or a HA-MAVS plasmid together with wild-type NS1 (Lane 2), truncated NS1 N (Δ74–230, Lane 3) or C (Δ1–73, Lane 4). Next, IP with HA antibody was performed and the presence of the NS1 protein was detected by a NS1 polyclonal antibody (**B**). Moreover, 293T cells were transiently transfected with MAVS-FLAG (Lanes 1–3), or empty vector expressing FLAG tag (Lane 4), along with either wild-type NS1-HA (WT), or the NS1 mutants (R38A/K41A and E96A/E97A), followed by IP using FLAG antibody (**C**). Expression of RIG-I in human 293T cells and chicken DF1 cells transfected with empty vector (EV) or wild-type NS1-HA (NS1) was validated by using RIG-I specific antibody (**D**). The interaction between NS1 and MAVS was examined in chicken DF1 cells co-transfected with MAVS plasmid and either NS1-FLAG (NS1) or empty FLAG tag plasmid (EV), followed by IP procedure with FLAG antibody (**E**). Wild-type A549 cells and its paired RIG-I^−/−^ (RIG-I KO) cells were infected with 0.5 MOI of recombinant PR8 viruses expressing FLAG tagged NS1 (NS1) or mock infected (−), followed by IP using FLAG antibody. MAVS specific antibody was applied to determine the level of endogenous MAVS. RIG-I specific antibody was used to examine the level of RIG-I in wild-type A549 and RIG-I KO cells (**F**).

Next, we explored which domains or residues in NS1 are required for the NS1-MAVS interaction. First, plasmids expressing NS1 deletion mutants were constructed by deleting the N-terminal RNA-binding domain (C; ∆1–73) or C-terminal effector domains (N; ∆74–230) (Figure 2A). Surprisingly, the deletion of either one of these two NS1 domains did not affect the ability of NS1 to bind MAVS, indicating that multiple motifs in NS1 mediate its interaction with MAVS (Figure 2B). Furthermore, we investigated whether NS1 directly associates with MAVS or indirectly influences its functions via other cellular proteins such as RIG-I and its activator, TRIM25. Both of these proteins could possibly facilitate the NS1-MAVS interaction, as previous studies have shown that the interaction between NS1 and RIG-1 or TRIM25 is essential for downstream activation of IRF3 through MAVS [24,25]. Therefore, two NS1 mutants, the RNA-binding mutant R38A/K41A and the TRIM25-binding mutant E96A/E97A [25], were employed to verify this supposition. As shown in Figure 2C, these two NS1 mutants retained MAVS-binding ability, suggesting that RNA, RIG-I, and TRIM25 do not mediate the association between NS1 and MAVS. Consistent results were observed in chicken DF1 cells that are deficient in RIG-I expression (Figure 2D) [28]; the results in Figure 2E demonstrated NS1-MAVS association in the absence of RIG-I. Lastly, we used human A549 cells with knock out of RIGI-I expression (RIG-I^−/−^) [26] to further confirm the NS1-MAVS interaction. As shown in Figure 2F, RIG-I was detected in wild-type A549, while absent in RIG-I^−/−^ (RIG-I KO) cells. Consistent with result obtained from DF1 cells, the endogenous MAVS was pulled down by NS1-FLAG in influenza virus infected A549 RIG-I KO cells, indicating NS1 interacts with MAVS in a RIG-I-independent manner.

### 3.3. MAVS Enhances the Mitochondrial Targeting of NS1, and the TM Tegion of MAVS Is Essential for the NS1-MAVS Interaction

**Figure 3:**
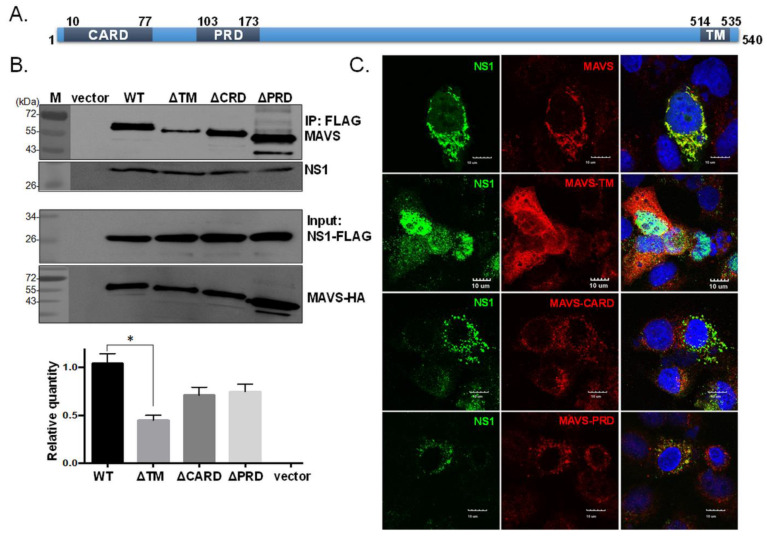
The transmembrane domain of MAVS is essential for its interaction with NS1. MAVS mutants with each functional domain deleted, i.e., the CARD-like domain (ΔCARD), proline-rich domain (ΔPRD), and transmembrane (ΔTM) region, were constructed and used to examine the region contributing to MAVS interaction with NS1 (**A**). Cells were co-transfected with NS1-FLAG plasmid with an empty vector (vector), wild-type MAVS (WT), or a MAVS mutant (ΔTM, ΔCARD, or ΔPRD). NS1 was pulled down by the FLAG antibody conjugated with beads, and the presence of MAVS was detected by Western blot analysis (**B**). Colocalization of NS1 with full-length or mutant forms of MAVS was assessed by IFA (**C**). The experiment was conducted three times. The intensity of the wild-type MAVS was arbitrarily set to 1, and the relative binding efficiency of the various MAVS mutants was estimated (**B**). One-way ANOVA was performed to analyze differences between means. * *p* < 0.05 indicated significance.

We next examined which domain in MAVS is important for the interaction with NS1. MAVS contains a proline-rich domain (PRD), a caspase recruitment domain (CARD), and a transmembrane domain (TM) (Figure 3A); the latter two domains are essential for MAVS signaling [29]. In Figure 3B,C, we found that the absence of the TM domain in MAVS significantly reduced its ability to interact with NS1, according to the IP and IFA results, suggesting that the TM region plays an important role in the NS1-MAVS interaction.

**Figure 4:**
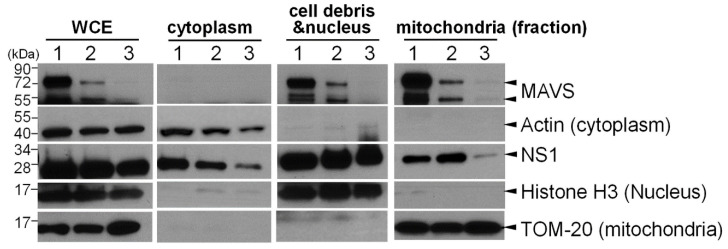
MAVS is important for NS1 translocation to mitochondria. Human 293T cells expressing high levels of MAVS (Lane 1), stably bearing siRNA containing scrambled sequences (negative control, Lane 2) or siRNA targeting MAVS (Lane 3) were transfected with a plasmid expressing FLAG-NS1. Whole cell extracts (WCE) were prepared, and fractions of the cytoplasm and mitochondria were further extracted from the nuclear fraction. The level of FLAG-NS1 and MAVS in each fraction was detected by Western blot analysis. Actin, TOM-20, and histone H3 marked the cytoplasmic, mitochondrial, or nuclear factions, respectively.

Additionally, since the TM region is essential for the transportation of MAVS to the outer membrane of mitochondria [30], we also investigated whether MAVS contributes to the translocation of NS1 to mitochondria. The subcellular distribution of NS1 was examined in 293T cells in which MAVS was transiently overexpressed (Figure 4, Lane 1) or suppressed by siRNA transfection (Figure 4, Lane 3). Nuclear, cytoplasmic, and mitochondrial fractions were prepared from the treated cells, and specific markers were used to identify subcellular locations, including histone H3 (nucleus), β-actin (cytoplasm), and TOM-20 (mitochondria). Reduced MAVS expression strongly correlated with reduced localization of NS1 to mitochondria (Figure 4, Lane 3). Nevertheless, the overexpression of MAVS did not further enhance the translocation of NS1 to mitochondria (Figure 4, Lane 1). Collectively, these data implied that MAVS is a critical factor for NS1 targeting to mitochondria and that the endogenous level of MAVS is sufficient for the recruitment of NS1 to mitochondria.

### 3.4. NS1 Decreases MAVS-Induced IFN Expression

**Figure 5:**
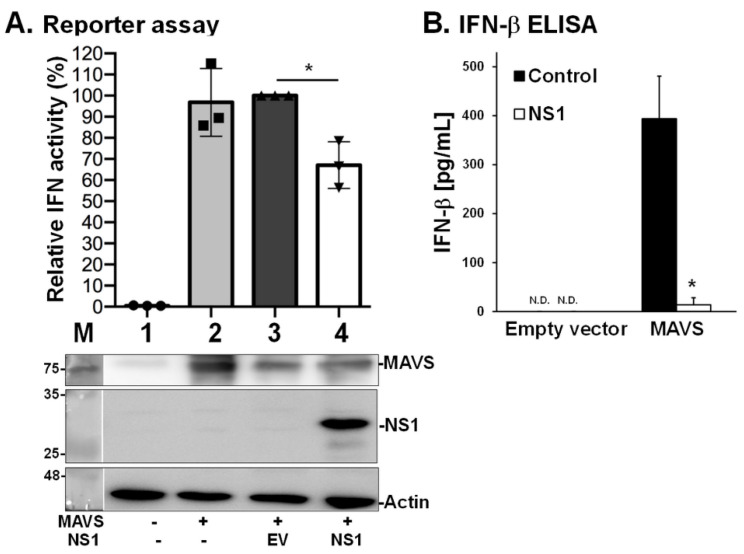
NS1 suppresses MAVS-induced Type I IFN expression. The effect of NS1 on MAVS-mediated IFN induction was estimated by IFN-β promoter activity using a reporter assay (**A**) and by IFN-β production using ELISA (**B**). These methods have been previously described [29]. Human 293T cells were transfected with a pIFN-GFP/CAT reporter plasmid and MAVS (Samples 2–4) together with the NS1 construct (PR8 NS1, Sample 4) or empty vector (EV, Sample 3). Whole cell lysates were harvested for detection of luciferase activity (reporter assay) and protein levels (Western blot analysis). Fold induction by the IFN promoter was determined by measuring normalized reporter activity and comparing it with that of the cells transfected with an empty vector. The experiment was conducted in triplicate. Error bars indicate standard deviation. One-way ANOVA was performed to analyze differences between means. * *p* < 0.05 indicated significance.

As MAVS serves as a potent adaptor for Type I IFN responses [29], we also explored whether the interaction between NS1 and MAVS interferes with the ability of MAVS to induce Type I IFN. We used two different assays to address this question [24], including a reporter assay to estimate IFN-β promoter activity (Figure 5A) and an ELISA to measure levels of IFN-β (Figure 5B). The data showed that NS1 significantly inhibited the MAVS-mediated signaling pathway, leading to a reduction in IFN-β levels. These results suggest that the interaction between NS1 and MAVS is involved in the disruption of host innate immune responses.

## 4. Discussion

IAV has evolved to encode various multifunctional proteins due to its limited genetic content, and IAV NS1 is a typical example. Several studies have investigated cellular interaction partners with NS1 during IAV infection, and different strategies have specific strengths and weaknesses [14,30]. NS1 has various functions in different intracellular compartments [30,31,32]. According to previous studies, NS1 has been shown to localize to the nucleus in infected cells where NS1 interferes with the cellular pre-mRNA processing machinery of the host and thus limits the expression of host genes [18,33]. Through the effector domain in the C-terminus, the NS1 protein directly interacts with the 30 kDa subunit of the cleavage and polyadenylation specificity factor (CPSF30), which results in inhibition of host gene expression. This process inhibits the maturation of host pre-mRNA. The nuclear export of cellular mRNA, including that of IFN and IFN-stimulated genes, is also suppressed by this mechanism [18,34,35]. Moreover, the accumulation of NS1 in the cytoplasm has been shown to correlate with enhanced IFN antagonism in primary chicken fibroblasts [36], possibly due to the interaction of NS1 with cellular proteins involved in IFN signaling pathways [31]. These findings suggest that the subcellular distribution of NS1 is one of the key factors influencing the pathogenicity of IAV [36].

NS1 can localize in both the nucleus and cytoplasm, the ratio of its destination is altered depending on the infection duration, virus strain, and cell types [7,37,38]. Until recently, there was no evidence showing the organelles to which IAV NS1 localized. The dynamic cellular distribution of NS1 was described and measured in transfected cells [39]. Moreover, Tsai et al. used a membrane-permeable dye to track NS1 in live cells during IAV infection and found that IAV NS1 was targeted to mitochondria and then translocated into subnuclear domains [39]. In addition to this evidence of mitochondrial localization, previous data revealed an interaction between NS1 and components in the RIG-I/MAVS pathway, such as RIG-I and TRIM25 [24,25]. Therefore, we speculated that NS1 may associate with MAVS, which mainly localizes to the mitochondria, and plays an important role in facilitating RIG-I signals. In this study, we exploited protein overexpression and viral infection methods to test this hypothesis, and the mitochondrial localization of NS1 could be recapitulated in different cell lines (Figure 1). However, based on data from bimolecular fluorescence complementation (BiFC) assays, Sánchez-Aparicio et al. suggested that there is no interaction between NS1 and MAVS [40]. This observation might have been caused by the fusion of fluorescent proteins causing altered protein folding or other steric hindrances to protein-protein interaction. We found that there were multiple binding sites in NS1 capable of enabling the NS1-MAVS interaction, and both the N- and C-termini of NS1 could independently bind to MAVS (Figure 2). Similarly, the involvement of multiple binding sites in the intermolecular interaction has been shown in different RNA viruses, such as human immunodeficiency virus (HIV) and Zika virus (ZIKV). Very recently, by using co-immunoprecipitation, the involvement of ZIKV NS4A in countering the IFN was demonstrated. Through the interaction with both the N-terminal CARD-like (CL) domain and C-terminal TM domains of MAVS, ZIKV NS4A protein prevents RIG-I from binding MAVS that ultimately attenuates IFN production [41]. Moreover, Pollpeter et al. revealed the interaction of HIV reverse transcriptase (RT) with the host anti-viral protein APOBEC3G and further identified two discrete and non-overlapping regions (fragments spanning residues 65 to 132, and the region 30 to 42) of APOBEC3G that are required for the HIV RT binding [42].

It is known that mitochondria serve as central organelles for multiple signal transduction pathways which contribute to numerous crucial cellular processes, including cellular respiration, apoptosis, and innate antiviral immunity [43]. According to previous studies, MAVS mediates multiple innate immune responses against viral infection, including the stimulation of NF-κB and IRF3/7 signaling [44] and the direct interaction with tumor necrosis factor receptor-associated factors (TRAFs), leading to the activation of IκB kinase (IKK) and tank-binding kinase 1 (TBK1) [45]. In particular, the TM domain of MAVS mediates MAVS self-association [46], and this domain is critical for MAVS localization to the mitochondrial outer membrane and the induction of Type I IFN expression [44,46]. Our data suggested that the TM domain of MAVS is critical for NS1 mitochondria targeting. However, further experiments are required to address whether the NS1-MAVS interaction prevents MAVS from associating with its downstream signaling transducers, such as TRAFs and TBK1. Several lines of evidence demonstrated that MAVS connects mitochondria with antiviral innate immunity that involves structural rearrangement and with the aid of several cellular proteins, such as RIG-I, and caseinolytic peptidase B protein homolog (CLPB) [47]. It is likely that the NS1-MAVS interaction can occur independently of RIG-I as indicated in the immunoprecipitation assay by using the NS1 constructs that are defective in RIG-I binding ability (Figure 2C). However, considering MAVS dynamically associates with various cellular proteins, immunoprecipitation experiments using whole cell lysates may not detect direct interaction between NS1 and MAVS, and likely additional cellular factors bridge the connection between NS1 and MAVS.

Accumulated evidence demonstrated that IAV has evolved multiple strategies to evade mitochondria-mediated innate immunity. In addition to NS1, PB1-F2, another non-structural protein encoded by some strains of influenza virus, is known to harbor a proapoptotic property specifically in immune cells, translocates to mitochondria and interacts with MAVS. The PB1-F2 recruitment to mitochondria further dampens IFN production by decreasing the mitochondrial membrane potential [48,49]. Moreover, nonstructural proteins of other RNA viruses, such as respiratory syncytial virus (RSV) [50], rotavirus [51], Zika virus (ZIKV) [52] and Dengue virus (DENV) [53], have been found to interact with MAVS and inhibit host antiviral responses using different strategies. The RSV NS1 protein associates with MAVS to negatively regulate the RIG-I-MAVS interaction, leading to interference of Type I IFN production [50]. The C-terminus of rotavirus NS1 binds to MAVS, and this interaction results in the degradation of MAVs and the subsequent suppression of NF-κB and IRF3 signaling [51]. ZIKV NS4A interacts with the N-terminal domain of MAVS and competes with RIG-I for binding to MAVS, a process similar to that of DENV NS4A [52,53]. Because viral RNA is sensed by multiple nucleic acid sensors in host cells to drive innate immune responses [54,55], MAVS in the RIG-I pathway has been targeted by many RNA viruses as a common mechanism to antagonize innate immune responses.

## 5. Conclusions

Our data suggest a novel function of IAV NS1 in interfering with host innate immunity by binding to the mitochondrial protein MAVS. The presence of mitochondrial localization of MAVS facilitates the mitochondrial localization of NS1, and this interaction downregulates MAVS-mediated antiviral responses, such as that leading to Type I IFN production. The detailed molecular mechanism of whether the binding of NS1 to MAVS leads to steric hindrance preventing RIG-I signaling remains to be clarified. Finally, the significance of IAV NS1 in viral replication and pathogenesis also requires further investigation in the future.

## Supplementary Materials

The following are available online at https://www.mdpi.com/article/10.3390/v13101909/s1, Figure S1: Cellular distribution of NS1 and MAVS in transfected cells. A549 cells were transfected by the plasmid expressing NS1 or MAVS. Cellular distribution and association of NS1 and MAVS were examined by IFA. Staining of 4’,6-diamidino-2-phenylindole (DAPI), shown as blue, was used to indicate the location of nucleus; Figure S2: Proportion of NS1 protein colocalized with MAVS in transfected cells. Colocalization of NS1 with MAVS were estimated by two types of software, namely ImageJ and the Olympus cellSens Dimension as labelled on the top of each image. The percentage of NS1-MAVS colocalization, as indicated in white color, was measured by ImageJ. Moreover, the entire scatterplot of NS1 and MAVS localization was also plotted by the Olympus 2 cellSens Dimension package software and the degree of overlap between the two profiles was further analyzed by Pearson’s correlation coefficient R(r). Six of representative cells were shown; Table S1: Colocalization of NS1 and MAVS.

## References

[1] Krug R.M., Etkind P.R. (1973). Cytoplasmic and nuclear virus-specific proteins in influenza virus-infected MDCK cells. Virology.

[2] Suarez D.L., Perdue M.L. (1998). Multiple alignment comparison of the non-structural genes of influenza A viruses. Virus Res..

[3] Hale B.G., Randall R.E., Ortin J., Jackson D. (2008). The multifunctional ns1 protein of influenza A viruses. J. Gen. Virol..

[4] Qian X.Y., Chien C.Y., Lu Y., Montelione G.T., Krug R.M. (1995). An amino-terminal polypeptide fragment of the influenza virus NS1 protein possesses specific RNA-binding activity and largely helical backbone structure. RNA.

[5] Wang X., Basler C.F., Williams B.R., Silverman R.H., Palese P., Garcia-Sastre A. (2002). Functional replacement of the carboxy-terminal two-thirds of the influenza A virus NS1 protein with short heterologous dimerization domains. J. Virol..

[6] Greenspan D., Palese P., Krystal M. (1988). Two nuclear location signals in the influenza virus NS1 nonstructural protein. J. Virol..

[7] Li Y., Yamakita Y., Krug R.M. (1998). Regulation of a nuclear export signal by an adjacent inhibitory sequence: The effector domain of the influenza virus NS1 protein. Proc. Natl. Acad. Sci. USA.

[8] Murayama R., Harada Y., Shibata T., Kuroda K., Hayakawa S., Shimizu K., Tanaka T. (2007). Influenza A virus non-structural protein 1 (NS1) interacts with cellular multifunctional protein nucleolin during infection. Biochem. Biophys. Res. Commun..

[9] Han H., Cui Z.-Q., Wang W., Zhang Z.-P., Wei H.-P., Zhou Y.-F., Zhang X.-E. (2010). New regulatory mechanisms for the intracellular localization and trafficking of influenza A virus NS1 protein revealed by comparative analysis of A/PR/8/34 and A/Sydney/5/97. J. Gen. Virol..

[10] Melen K., Kinnunen L., Fagerlund R., Ikonen N., Twu K.Y., Krug R.M., Julkunen I. (2007). Nuclear and nucleolar targeting of influenza A virus NS1 protein: Striking differences between different virus subtypes. J. Virol..

[11] Volmer R., Mazel-Sanchez B., Volmer C., Soubies S.M., Guerin J.-L. (2010). Nucleolar localization of influenza a NS1: Striking differences between mammalian and avian cells. Virol. J..

[12] Garaigorta U., Falcon A.M., Ortin J. (2005). Genetic analysis of influenza virus NS1 gene: A temperature-sensitive mutant shows defective formation of virus particles. J. Virol..

[13] Sato Y., Yoshioka K., Suzuki C., Awashima S., Hosaka Y., Yewdell J., Kuroda K. (2003). Localization of influenza virus proteins to nuclear dot 10 structures in influenza virus-infected cells. Virology.

[14] Raman S.N.T., Zhou Y. (2016). Networks of host factors that interact with NS1 protein of influenza A virus. Front. Microbiol..

[15] Garcia-Sastre A., Egorov A., Matassov D., Brandt S., Levy D.E., Durbin J.E., Palese P., Muster T. (1998). Influenza A virus lacking the NS1 gene replicates in interferon-deficient systems. Virology.

[16] Talon J., Horvath C.M., Polley R., Basler C.F., Muster T., Palese P., Garcia-Sastre A. (2000). Activation of interferon regulatory factor 3 is inhibited by the influenza A virus NS1 protein. J. Virol..

[17] Wang X., Li M., Zheng H., Muster T., Palese P., Beg A.A., Garcia-Sastre A. (2000). Influenza A virus NS1 protein prevents activation of nf-kappab and induction of alpha/beta interferon. J. Virol..

[18] Nemeroff M.E., Barabino S.M., Li Y., Keller W., Krug R.M. (1998). Influenza virus NS1 protein interacts with the cellular 30 kDa subunit of cpsf and inhibits 3’end formation of cellular pre-mRNAs. Mol. Cell.

[19] Fortes P., Beloso A., Ortin J. (1994). Influenza virus NS1 protein inhibits pre-mRNA splicing and blocks mRNA nucleocytoplasmic transport. EMBO J..

[20] Lu Y., Qian X.Y., Krug R.M. (1994). The influenza virus NS1 protein: A novel inhibitor of pre-mRNA splicing. Genes Dev..

[21] Min J.-Y., Krug R.M. (2006). The primary function of RNA binding by the influenza A virus NS1 protein in infected cells: Inhibiting the 2’-5’ oligo (A) synthetase/RNase l pathway. Proc. Natl. Acad. Sci. USA.

[22] Min J.Y., Li S., Sen G.C., Krug R.M. (2007). A site on the influenza A virus NS1 protein mediates both inhibition of PKR activation and temporal regulation of viral RNA synthesis. Virology.

[23] Rajsbaum R., Albrecht R.A., Wang M.K., Maharaj N.P., Versteeg G.A., Nistal-Villan E., Garcia-Sastre A., Gack M.U. (2012). Species-specific inhibition of RIG-I ubiquitination and ifn induction by the influenza A virus NS1 protein. PLoS Pathog..

[24] Mibayashi M., Martinez-Sobrido L., Loo Y.-M., Cardenas W.B., Gale M., Garcia-Sastre A. (2007). Inhibition of retinoic acid-inducible gene I-mediated induction of beta interferon by the NS1 protein of influenza A virus. J. Virol..

[25] Gack M.U., Albrecht R.A., Urano T., Inn K.-S., Huang I.-C., Carnero E., Farzan M., Inoue S., Jung J.U., Garcia-Sastre A. (2009). Influenza A virus NS1 targets the ubiquitin ligase TRIM25 to evade recognition by the host viral RNA sensor RIG-I. Cell Host Microbe.

[26] Schuberth-Wagner C., Ludwig J., Bruder A.K., Herzner A.-M., Zillinger T., Goldeck M., Schmidt T., Schmid-Burgk J.L., Kerber R., Wolter S. (2015). A conserved histidine in the RNA sensor RIG-I controls immune tolerance to N1-2’o-methylated self RNA. Immunity.

[27] Heaton N.S., Moshkina N., Fenouil R., Gardner T.J., Aguirre S., Shah P.S., Zhao N., Manganaro L., Hultquist J.F., Noel J. (2016). Targeting viral proteostasis limits influenza virus, hiv, and dengue virus infection. Immunity.

[28] Lee S.B., Park Y.H., Chungu K., Woo S.J., Han S.T., Choi H.J., Rengaraj D., Han J.Y. (2020). Targeted knockout of mda5 and tlr3 in the df-1 chicken fibroblast cell line impairs innate immune response against RNA ligands. Front. Immunol..

[29] Varga Z.T., Ramos I., Hai R., Schmolke M., Garcia-Sastre A., Fernandez-Sesma A., Palese P. (2011). The influenza virus protein pb1-f2 inhibits the induction of type i interferon at the level of the mavs adaptor protein. PLoS Pathog..

[30] Tawaratsumida K., Phan V., Hrincius E.R., High A.A., Webby R., Redecke V., Hacker H. (2014). Quantitative proteomic analysis of the influenza A virus nonstructural proteins NS1 and NS2 during natural cell infection identifies pact as an NS1 target protein and antiviral host factor. J. Virol..

[31] Ayllon J., Garcia-Sastre A. (2015). The NS1 protein: A multitasking virulence factor. Curr. Top. Microbiol. Immunol..

[32] Nogales A., Rodriguez L., DeDiego M.L., Topham D.J., Martinez-Sobrido L. (2017). Interplay of PA-X and NS1 proteins in replication and pathogenesis of a temperature-sensitive 2009 pandemic H1N1 influenza A virus. J. Virol..

[33] Twu K.Y., Noah D.L., Rao P., Kuo R.-L., Krug R.M. (2006). The cpsf30 binding site on the NS1A protein of influenza A virus is a potential antiviral target. J. Virol..

[34] Ramos I., Carnero E., Bernal-Rubio D., Seibert C.W., Westera L., Garcia-Sastre A., Fernandez-Sesma A. (2013). Contribution of double-stranded RNA and CPSF30 binding domains of influenza virus NS1 to the inhibition of type I interferon production and activation of human dendritic cells. J. Virol..

[35] Rodriguez L., Nogales A., Iqbal M., Perez D.R., Martinez-Sobrido L. (2018). Identification of amino acid residues responsible for inhibition of host gene expression by influenza A H9N2 NS1 targeting of CPSF30. Front. Microbiol..

[36] Keiner B., Maenz B., Wagner R., Cattoli G., Capua I., Klenk H.D. (2010). Intracellular distribution of NS1 correlates with the infectivity and interferon antagonism of an avian influenza virus (H7N1). J. Virol..

[37] Li Y., Lu X., Li J., Berube N., Giest K.-L., Liu Q., Anderson D.H., Zhou Y. (2010). Genetically engineered, biarsenically labeled influenza virus allows visualization of viral NS1 protein in living cells. J. Virol..

[38] Mok B.-W., Liu H., Chen P., Liu S., Lau S.-Y., Huang X., Liu Y.-C., Wang P., Yuen K.-Y., Chen H. (2017). The role of nuclear NS1 protein in highly pathogenic H5N1 influenza viruses. Microbes Infect..

[39] Tsai C.F., Lin H.Y., Hsu W.L., Tsai C.H. (2017). The novel mitochondria localization of influenza A virus NS1 visualized by flash labeling. FEBS Open Bio..

[40] Sanchez-Aparicio M.T., Ayllon J., Leo-Macias A., Wolff T., Garcia-Sastre A. (2017). Subcellular localizations of RIG-I, TRIM25, and MAVS complexes. J. Virol..

[41] Hu Y., Dong X., He Z., Wu Y., Zhang S., Lin J., Yang Y., Chen J., An S., Yin Y. (2019). Zika virus antagonizes interferon response in patients and disrupts RIG-I-mavs interaction through its CARD-TM domains. Cell Biosci..

[42] Pollpeter D., Parsons M., Sobala A.E., Coxhead S., Lang R.D., Bruns A.M., Papaioannou S., McDonnell J.M., Apolonia L., Chowdhury J.A. (2018). Deep sequencing of HIV-1 reverse transcripts reveals the multifaceted antiviral functions of APOBEC3G. Nat. Microbiol..

[43] Koshiba T. (2013). Mitochondrial-mediated antiviral immunity. Biochim. Biophys. Acta.

[44] Seth R.B., Sun L., Ea C.K., Chen Z.J. (2005). Identification and characterization of MAVS, a mitochondrial antiviral signaling protein that activates NF-KB and IRF 3. Cell.

[45] Shi Y., Yuan B., Qi N., Zhu W., Su J., Li X., Qi P., Zhang D., Hou F. (2015). An autoinhibitory mechanism modulates MAVS activity in antiviral innate immune response. Nat. Commun..

[46] Tang E.D., Wang C.-Y. (2009). Mavs self-association mediates antiviral innate immune signaling. J. Virol..

[47] Yoshinaka T., Kosako H., Yoshizumi T., Furukawa R., Hirano Y., Kuge O., Tamada T., Koshiba T. (2019). Structural basis of mitochondrial scaffolds by prohibitin complexes: Insight into a role of the Coiled-Coil region. iScience.

[48] Yoshizumi T., Ichinohe T., Sasaki O., Otera H., Kawabata S.-I., Mihara K., Koshiba T. (2014). Influenza A virus protein PB1-F2 translocates into mitochondria via TOM40 channels and impairs innate immunity. Nat. Commun..

[49] Varga Z.T., Grant A., Manicassamy B., Palese P. (2012). Influenza virus protein PB1-F2 INHIBITS the induction of type I interferon by binding to MAVS and decreasing mitochondrial membrane potential. J. Virol..

[50] Boyapalle S., Wong T., Garay J., Teng M., Juan-Vergara H.S., Mohapatra S., Mohapatra S. (2012). Respiratory syncytial virus NS1 protein colocalizes with mitochondrial antiviral signaling protein mavs following infection. PLoS ONE.

[51] Nandi S., Chanda S., Bagchi P., Nayak M.K., Bhowmick R., Chawla-Sarkar M. (2014). Mavs protein is attenuated by rotavirus nonstructural protein 1. PLoS ONE.

[52] Ma J., Ketkar H., Geng T., Lo E., Wang L., Xi J., Sun Q., Zhu Z., Cui Y., Yang L. (2018). Zika virus non-structural protein 4A blocks the RLR-MAVS signaling. Front. Microbiol..

[53] He Z., Zhu X., Wen W., Yuan J., Hu Y., Chen J., An S., Dong X., Lin C., Yu J. (2016). Dengue virus subverts host innate immunity by targeting adaptor protein MAVS. J. Virol..

[54] Jensen S., Thomsen A.R. (2012). Sensing of RNA viruses: A review of innate immune receptors involved in recognizing RNA virus invasion. J. Virol..

[55] Chow K.T., Gale M., Loo Y.-M. (2018). RIG-I and other RNA sensors in antiviral immunity. Annu. Rev. Immunol..

