# Supplementary material for: Interaction between NS1 and Cellular MAVS Contributes to NS1 Mitochondria Targeting
Source: Viruses. 2021 Sep 23;13(10):1909. doi: 10.3390/v13101909 (PMC8537625; doi:10.3390/v13101909)
Supplement: Supplementary file 1 [file viruses-13-01909-s001.zip › viruses-1375383-supplementary.pdf]

viruses

MDPI

Supplementary file

Interaction between NS1 and Cellular MAVS Contributes to NS1 Mitochondria Targeting

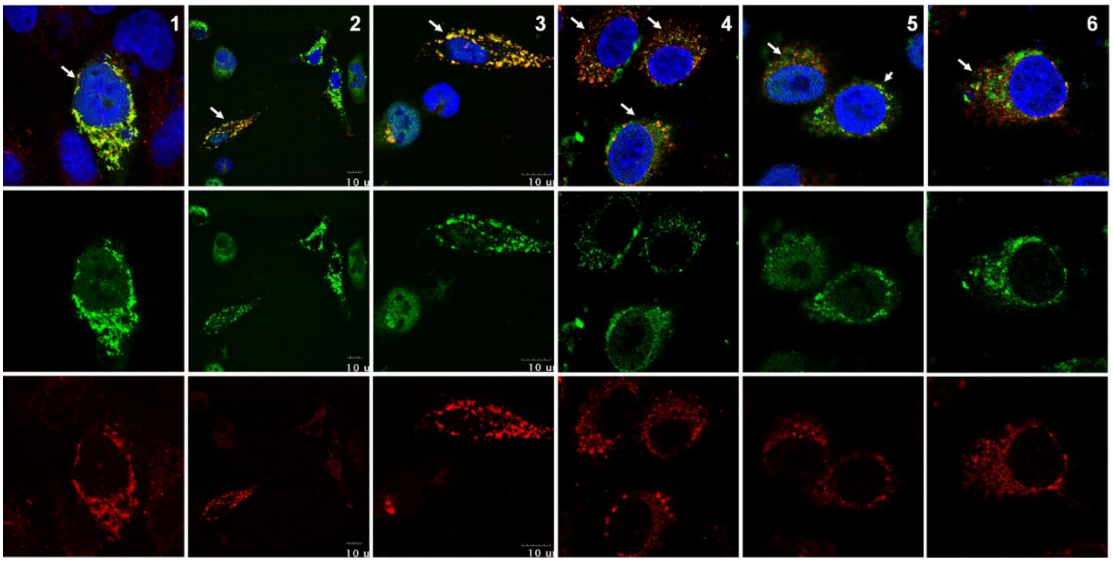

Figure S1. Cellular distribution of NS1 and MAVS in transfected cells. A549 cells were transfected by the plasmid expressing NS1 or MAVS. Cellular distribution and association of NS1 and MAVS were examined by IFA. Staining of 4',6-diamidino-2-phenylindole (DAPI), shown as blue, was used to indicate the location of nucleus.

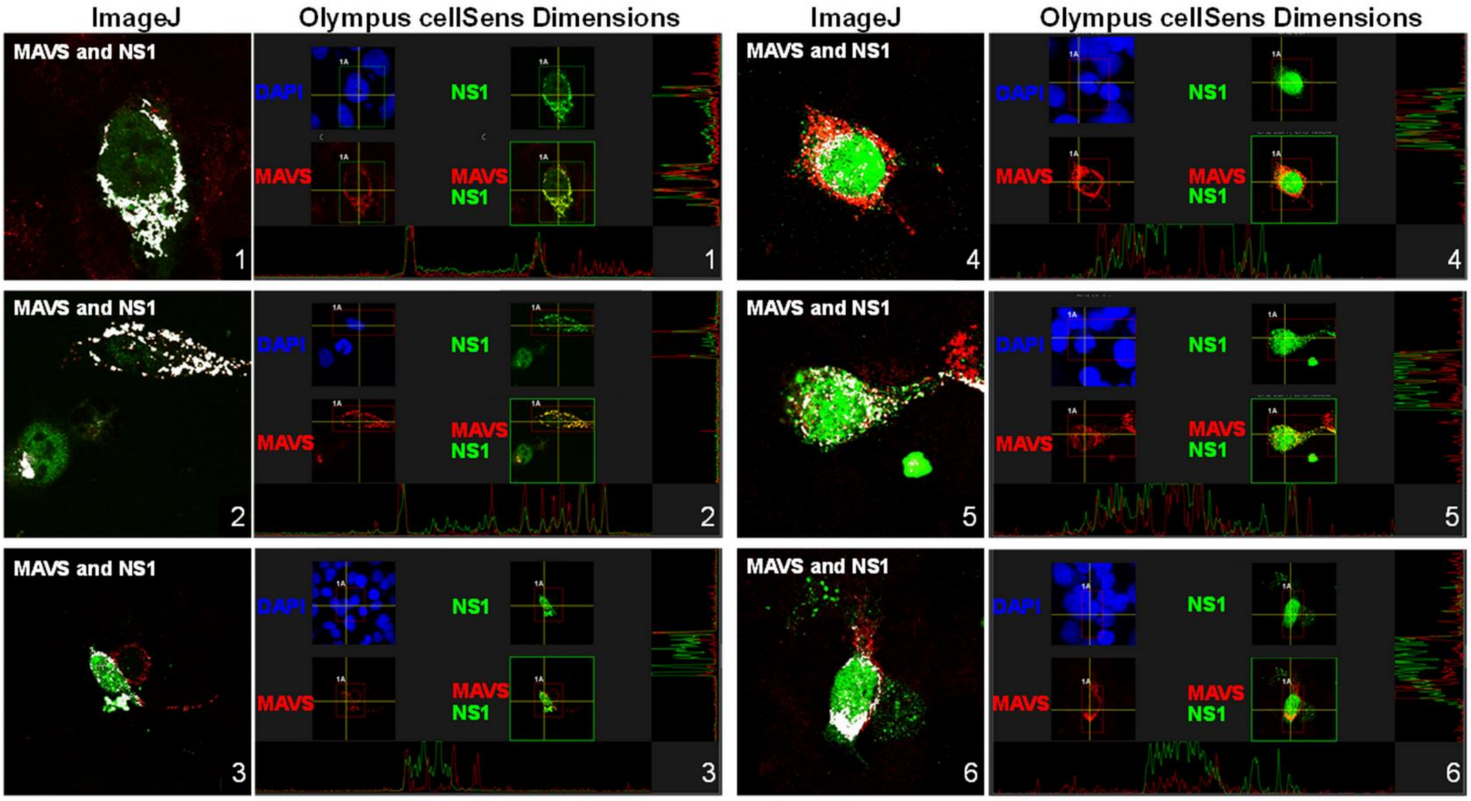

Figure S2. Proportion of NS1 protein colocalized with MAVS in transfected cells. Colocalization of NS1 with MAVS were estimated by two types of software, namely ImageJ and the Olympus cellSens Dimension as labelled on the top of each image. The percentage of NS1-MAVS colocalization, as indicated in white color, was measured by ImageJ. Moreover, the entire scatterplot of NS1 and MAVS localization was also plotted by the Olympus 2 cellSens Dimension package software and the degree of overlap between the two profiles was further analyzed by Pearson's correlation coefficient R(r). Six of representative cells were shown;.

Table S1. Colocalization of NS1 and MAVS.

	Colocalization/NS1	
**Cell**	**ImageJ**	**Pearson's Correlation Coefficient (r)** [a]
**1**	**67.6%**	**0.733**
**2**	**27.1%**	**0.306**
**3**	**60.5%**	**0.673**
**4**	**46.4%**	**0.287**
**5**	**27.5%**	**0.628**
**6**	**43%**	**0.125**

[a] the scatterplot of NS1 and MAVS localization was plotted by the Olympus cellSens Dimension package software.
